# Nomogram predicted survival of patients with adenocarcinoma of esophagogastric junction

**DOI:** 10.1186/s12957-015-0613-7

**Published:** 2015-06-10

**Authors:** Zhangjian Zhou, Hao Zhang, Zisen Xu, Wenhan Li, Chengxue Dang, Yongchun Song

**Affiliations:** Division of Surgical Oncology, The First Affiliated Hospital, Xi’an Jiaotong University, 277 W. Yanta Road, Xi’an, 710061, Shaanxi China

**Keywords:** Adenocarcinoma of esophagogastric junction, Nomogram, Predictor, Survival

## Abstract

**Background:**

The aim of this study is to develop a prognostic nomogram for patients with adenocarcinoma of esophagogastric junction and compare its predictive accuracy with the traditional tumor-node-metastasis (TNM) malignant staging system.

**Methods:**

Patients from the Surveillance, Epidemiology, and End Results Program (from 1988 to 2011) and the First Affiliated Hospital of Xi’an Jiaotong University (from 2005 to 2010) were collected retrospectively. Preselected multiple potential interactions were tested irrespective of significance as nomogram parameters. And the Harrell’s C-index was used to estimate the accuracy of the nomogram system. Model validation was performed using bootstrap to quantify our modeling strategy.

**Results:**

In our study, six clinical associated factors (age, sex, depth of invasion, metastasized lymph nodes, examined lymph nodes, histological grade) were evaluated in the nomogram. In the training set, the nomogram exhibited superior discrimination power compared with the American Joint Committee on Cancer (AJCC) TNM classification (Harrell’s C-index, 0.69 and 0.63, respectively). Calibration of the nomogram predicted survival was similar to the actual overall survival. In the validation set, the discrimination of nomogram was also better than the AJCC TNM staging system (C-index, 0.75 and 0.65, respectively), and the calibration of nomogram predicted survival was within a 10 % margin of actual overall survival.

**Conclusions:**

Based on the patients with adenocarcinoma of esophagogastric junction from a Western and an Eastern database, the nomogram provided significantly improved discrimination than the traditional AJCC TNM classification and also provided an accurate individualized prediction of the survival.

**Electronic supplementary material:**

The online version of this article (doi:10.1186/s12957-015-0613-7) contains supplementary material, which is available to authorized users.

## Background

In Western countries, there has been a dramatic increase in the incidence of adenocarcinomas of the esophagogastric junction (AEG). But in Eastern countries, they have not experienced such an increase [1–4]. The definition of AEG was confused until the recent Union for International Cancer Control (UICC) and American Joint Committee on Cancer (AJCC) tumor-node-metastasis (TNM) classification of malignant tumors had been published. Based on UICC classification, a tumor with an epicenter within 5 cm of the esophagogastric junction (EGJ) and extension into the esophagus is classified and staged according to the esophageal scheme; while tumors with an epicenter greater than 5 cm from the EGJ or those within 5 cm of the EGJ without extension into the esophagus were classified and staged according to the gastric carcinoma scheme [5]. There are many surgical approaches for AEG as a junctional cancer. Previously, some surgeons treat it like distal esophageal cancer and some others are more likely to treat it like proximal gastric cancer. This also means that they treat AEG according to different approaches, transhiatal, transthoracic, or transthoracoabdominal [6, 7]. Of this special anatomic location, the disparate surgical therapeutic regimen may affect the overall survival. The traditional TNM classification stratified the resectable AEG into three large groupings in terms of the pathologic depth of invasion, the number of lymph node metastasized, and the differentiation of the tumor. Nevertheless, it did not focus on other clinical features, such as age, sex, the size of the tumor, and the number of examined lymph node, which could be considered as the predicting factors that affect individualized survival. Therefore, a better evaluation system including demographic characteristics, pathological features, and treatment factors is required to make more accurate survival prediction. The nomogram is a statistics-based tool that provides the overall probability of a specific outcome. For many cancers, nomograms are proved to be more accurate in predicting individualized survival when compared with the traditional TNM staging systems [8–11]. It has been recently proposed as an alternative or even as a new standard.

Although some researches have estimated the overall survival of carcinomas of esophagus and stomach, adenocarcinoma of the esophagogastric junction, a newly redefined individual adenocarcinoma, has not been analyzed independently [12, 13]. The purpose of this study is to develop a prognostic nomogram for patients with AEG that are concerned by both thoracic surgeons and general surgeons and compare its predictive accuracy with the traditional TNM staging system.

## Methods

### Patients

The Surveillance, Epidemiology, and End Results Program (SEER) of the National Cancer Institute is an authoritative source of information on cancer incidence and survival in the USA. Data collected include patient demographic information, pathological information, and survival information from 1988 to 2011. The inclusion criteria are the following: (1) patients with adenocarcinomas located in esophagogastric junction; (2) patients who underwent surgery and exact pathological details can be achieved; (3) patients without distant metastasis; (4) Patients diagnosed after 2004 (those who can get a more accurate pathologic data); and (5) ICD-O-3 code within the range of 8140–8147, 8210–8214, 8220–8221, 8255, 8260–8263, 8310, and 8480–8481.

Another data set was retrospectively collected from an Eastern medical center, The First Affiliated Hospital of Xi’an Jiaotong University, from January 2005 to March 2010. The eligibility criteria were the same as the inclusion criteria of SEER database. The retrospectively collected data of these patients included demographic parameters, histopathologic tumor characteristics, operation methods, and survival time.

### Statistical analysis

We set the patients from SEER database as the training set (*n* = 953) and set patients from Xi’an Jiaotong University as the validation set (*n* = 181). Categorical variables were grouped based on the classification scheme that clinical doctors were interested in, and the classified groups were made before further analyses. Age, as a continuous variable, was analyzed using restricted cubic splines by the knots of four. The results were compared using the *χ*^2^ test or Fisher’s exact test. Continuous variables were compared using the Student *t* test. Survival curves were depicted using the Kaplan-Meier method and compared using the log-rank test. Cox proportional hazard models were constructed to investigate multivariable relationships of covariates with survival. The preselected multiple potential interactions were tested irrespective of significance as nomogram parameters [14]. For discrimination, we used the Harrell’s C-index to make an accurate estimate of the nomogram system. Model validation was performed using bootstrap to quantify our modeling strategy and obtain a relatively unbiased estimate. All statistical tests were two-sided, and *P* values <0.05 were considered to be statistically significant. Statistical analyses were performed using SPSS 13.0 and R software version 3.1.0 (http://www.r-project.org) with the “rms” package.

## Results

### Demographic and pathological characteristics of patients

For the training set, there were 953 patients who fulfilled the inclusion criteria between 2004 and 2011. Approximately 80 % of the patients were male (*n* = 755) and the rest of the 198 patients were female. The age of patients ranged from 23 to 91 years (median was 66 years old). There were 766 (80.4 %) patients with 7 or more lymph nodes resected, and the average lymph nodes resection was 15.8 (range, 1 to 90). For the validation set, 181 patients fulfilled the inclusion criteria between 2005 and 2010. More than 80 % of patients were male (*n* = 157) and 13.3 % patients were female (*n* = 24). The age of patients ranged from 40 to 86 years (median was 64 years old). There were 154 (85.1 %) patients with 7 or more lymph nodes resected, and the average lymph nodes resection was 14.3 (range, 2 to 53). The details of patient characteristics are listed in Table 1.

**Table 1:** Demographic and clinic pathologic variables of the training and validation sets

Variable	Training set		Validation set	
	(*n* = 953)		(*n* = 181)	
	No. of Patients	Percent	No. of patients	Percent
Age(year)				
<50	100	10.5	10	5.5
50–60	218	22.9	55	30.4
60–70	286	30.0	76	42
≥70	349	36.6	40	22.1
Sex				
Male	755	79.2	157	86.7
Female	198	20.8	24	13.3
Depth of invasion				
T1	203	21.3	10	5.5
T2	478	50.2	17	9.4
T3	236	24.8	152	84
T4	36	3.8	2	1.1
Lymph node metastasis (*n*)				
0	421	44.2	53	29.3
1–2	211	22.1	42	23.2
3–6	177	18.6	40	22.1
7–15	114	12.0	40	22.1
≥15	30	3.1	6	3.3
Examined lymph node (*n*)				
<5	116	12.2	8	4.4
5–10	211	22.1	55	30.4
10–15	206	21.6	63	34.8
15–20	173	18.2	28	15.5
20–25	109	11.4	11	6.1
25–35	97	10.2	12	6.6
35–45	23	2.4	3	1.7
≥45	18	1.9	1	0.6
Grade				
G1 + G2	425	44.6	91	50.3
G3 + G4	528	55.4	90	49.7

### Overall survival of the training set

For the 953 patients in the training set, the average follow-up time was 25.5 months and the overall 5-year survival rate was 38 %. For the multivariate analysis, six clinical associated factors (age, sex, depth of invasion, metastasized lymph nodes, examined lymph nodes, histological grade) were evaluated regardless of the result of univariate analysis to improve the performance of the nomogram. The results of the multivariate analysis (Table 2) show that age, depth of invasion, the number of metastasized lymph nodes, and the number of examined lymph nodes were the independent prognostic factors that were significantly associated with overall survival (*P* < 0.001).

**Table 2:** Multivariate analysis of the training set

Variable	Hazard ratio	95 % CI	*P* value
Age(year)	1.030	1.022–1.039	<0.001
Sex			
Male	Ref		
Female	0.849	0.672–1.073	0.170
Depth of invasion			
T1	Ref		
T2	1.788	1.319–2.423	<0.001
T3	1.821	1.297–2.555	0.001
T4	2.565	1.541–4.269	<0.001
Lymph node metastasis (*n*)			
0	Ref		
1–2	1.723	1.329–2.233	<0.001
3–6	2.386	1.823–3.124	<0.001
7–15	3.542	2.573–4.875	<0.001
≥15 m	6.947	4.250–11.353	<0.001
Examined lymph node (*n*)			
<5	Ref		
5–10	0.789	0.582–1.070	0.127
10–15	0.748	0.547–1.025	0.071
15–20	0.676	0.483–0.945	0.022
20–25	0.493	0.331–0.734	0.001
25–35	0.480	0.320–0.719	<0.001
35–45	0.370	0.185–0.741	0.005
≥45	0.188	0.084–0.422	<0.001
Grade			
G1 + G2	Ref		
G3 + G4	1.084	0.894–1.315	0.412

### Nomogram

**Fig. 1:**
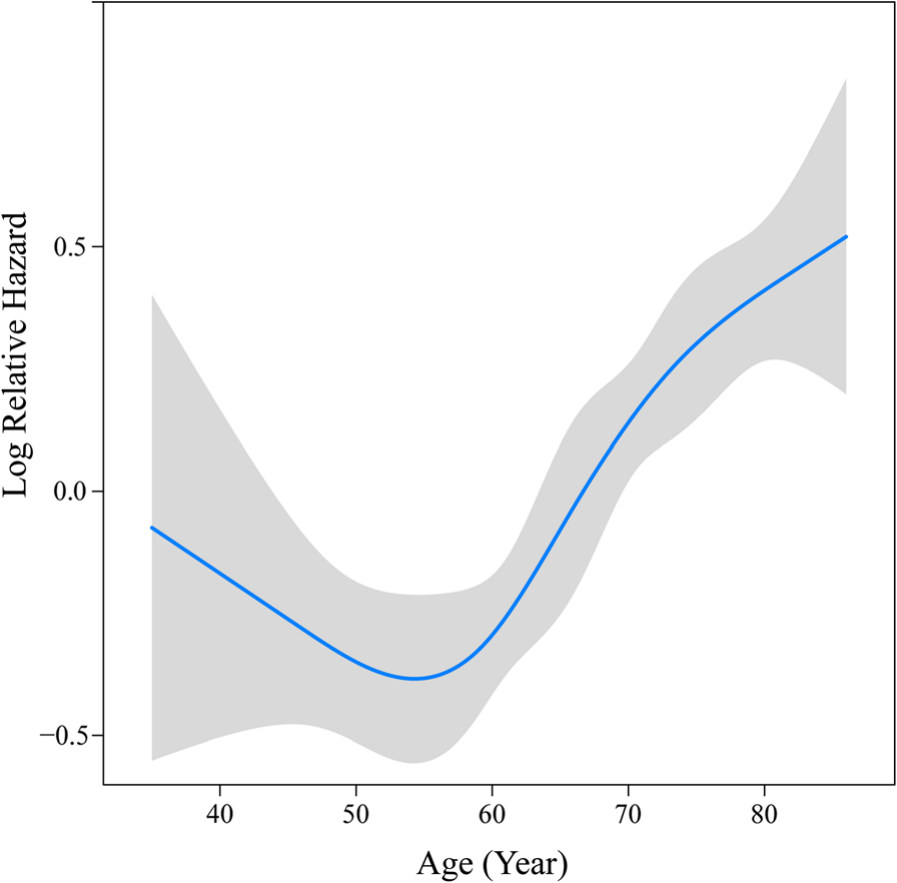
Transformation of continuous variables in univariate analysis using restricted cubic splines

**Fig. 2:**
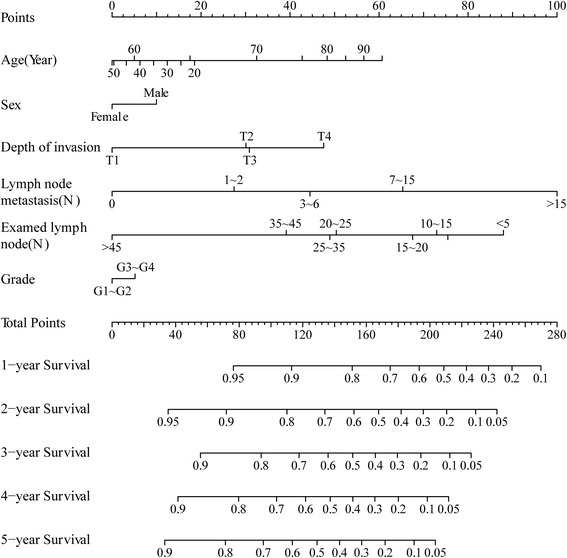
Nomogram predicted 1- to 5-year overall survival using six available clinical characteristics. To use the nomogram to calculate personal predicted survival, the patient’s age was located on the row labeled *Age* (*year*) and a straight line was drawn up to the row labeled *Points* to determine the corresponding points. This process was repeated for each of the remaining factors by drawing a straight line to the “Points” row to determine the points associated with each factor. After summarizing the total points, one located the appropriate total point number and drawn a straight line from this to the rows labeled *1*-*year survival*, *2*-*year survival*, *3*-*year survival*, *4*-*year survival*, and *5*-*year survival* to determine the patient’s predicted survival probability

For inclusion into the final nomogram model, effect of the continuous variable, age was explored using restricted cubic splines with four knots which made a satisfied sensitivity (Fig. 1). In the nomogram model, each factor from the multivariate Cox proportional hazard regression model was ascribed a weighted point total that implied a survival prognosis. For example, 65 years old was associated with 18 points, female was associated with zero point, depth of invasion (T2) was associated with 30 points, 5 metastasized lymph nodes was associated with 44 points, 36 examined lymph nodes was associated with 39 points, and well-differentiated adenocarcinoma was associated with zero point, so the total score points were 167. And each patient with a higher score had a worse prognosis. The final nomogram model to predict the survival (1- to 5-year overall survival) of AEG patients undergoing surgical resection is shown in Fig. 2.

**Fig. 3:**
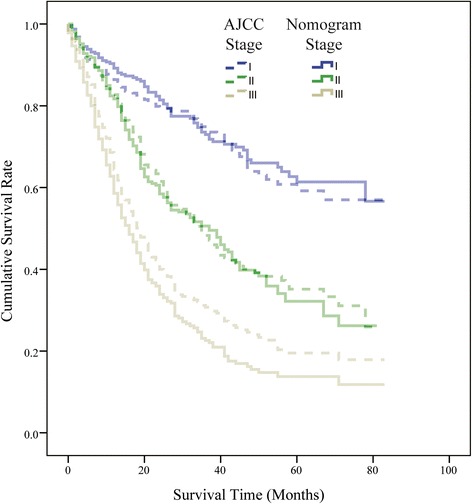
Kaplan-Meier survival curves of AJCC TNM stages and nomogram trisection stages of non-metastasis patients with AEG

Prognostic discrimination was performed by dividing the predicted survival probabilities into trisections in order to fit the AJCC TNM staging system of non-metastasis AEG. The results were then demonstrated with Kaplan-Meier plotting (Fig. 3, Additional file 1: Figure S1 and Additional file 2: Figure S2). In the training set, patients in the lowest trisection of predicted survival had a median recorded survival of 18.3 months, whereas those in the middle and top trisections had median recorded survival of 26.5 and 31.7 months, respectively. Meanwhile, the median recorded survival of three AJCC stages were 33.3 months for stage I, 25.5 months for stage II, and 20.4 months for stage III, respectively. The nomogram was able to stratify AEG patients into three distinct incremental 5-year survival prognostic groups (trisection I with 65 %, trisection II with 37 %, trisection III with 18 %). The 5-year overall survival of three nomogram stages was significantly different (*P* < 0.01). At the same time, the 5-year survival of three AJCC stages were 61 % of stage I, 41 % of stage II, and 24 % of stage III, respectively.

### Internal and external validation of the nomogram

**Fig. 4:**
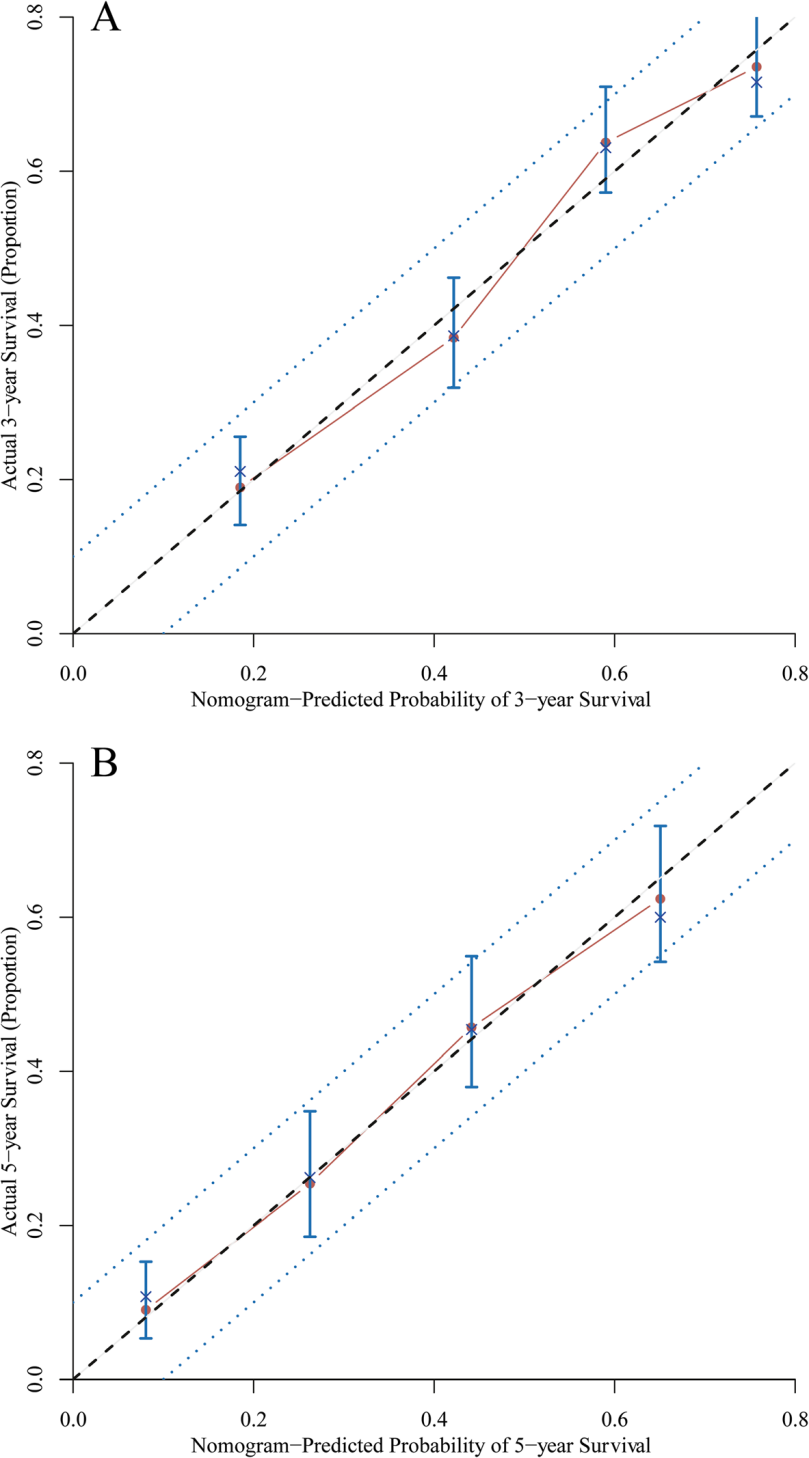
Calibration of the nomogram in the training set. Nomogram predicted probability of overall survival was plotted on the *x*-axis, actual overall survival was plotted on the *y*-axis and 95 % CIs measured by Kaplan-Meier analysis. All predictions lie within the 10 % margin of error (within the *blue dots* line). **a** Three-year survival. **b** Five-year survival

Predictive accuracy of the final nomogram model and AJCC model were measured by calculating the Harrell’s C-index. For the internal validation of the nomogram in the training set, the C-index was 0.69 (95 % confidence interval (CI), 0.66–0.72). It had a better discrimination compared with the AJCC TNM staging system which had a C-index of 0.63 (95 % CI, 0.61–0.66). Figure 4 shows the calibration plot of the nomogram of 3- and 5-year survival of the training set. As we could see, the predicted survival was corresponded closely with the actual survival and was always within the 10 % margin of error.

**Fig. 5:**
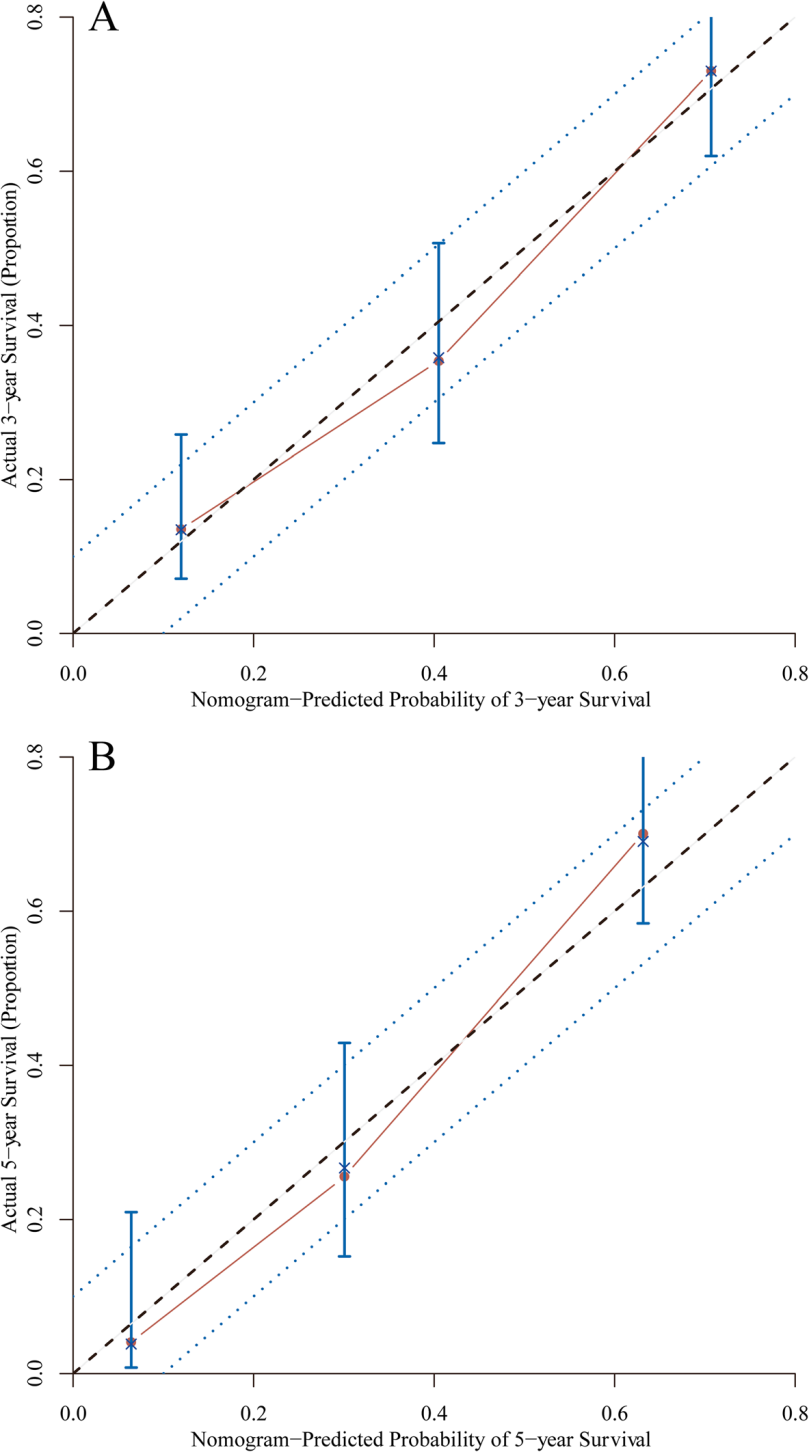
Calibration of the nomogram in the validation set. Nomogram predicted probability of overall survival was plotted on the *x*-axis, actual overall survival was plotted on the *y*-axis and 95 % CIs measured by Kaplan-Meier analysis. All predictions lie within the 10 % margin of error (within the *blue dots* line). **a** Three-year survival. **b** Five-year survival

For the external validation of nomogram in the validation set, the C-index was 0.75 (95 % CI, 0.71–0.79). Also, it was a better prognostic system than the traditional AJCC TNM staging classification which had a C-index of 0.65 (95 % CI, 0.61–0.69). Figure 5 shows the calibration plot of the nomogram of 3- and 5-year survival of the validation set.

### The relationship between nomogram and AJCC staging system

**Fig. 6:**
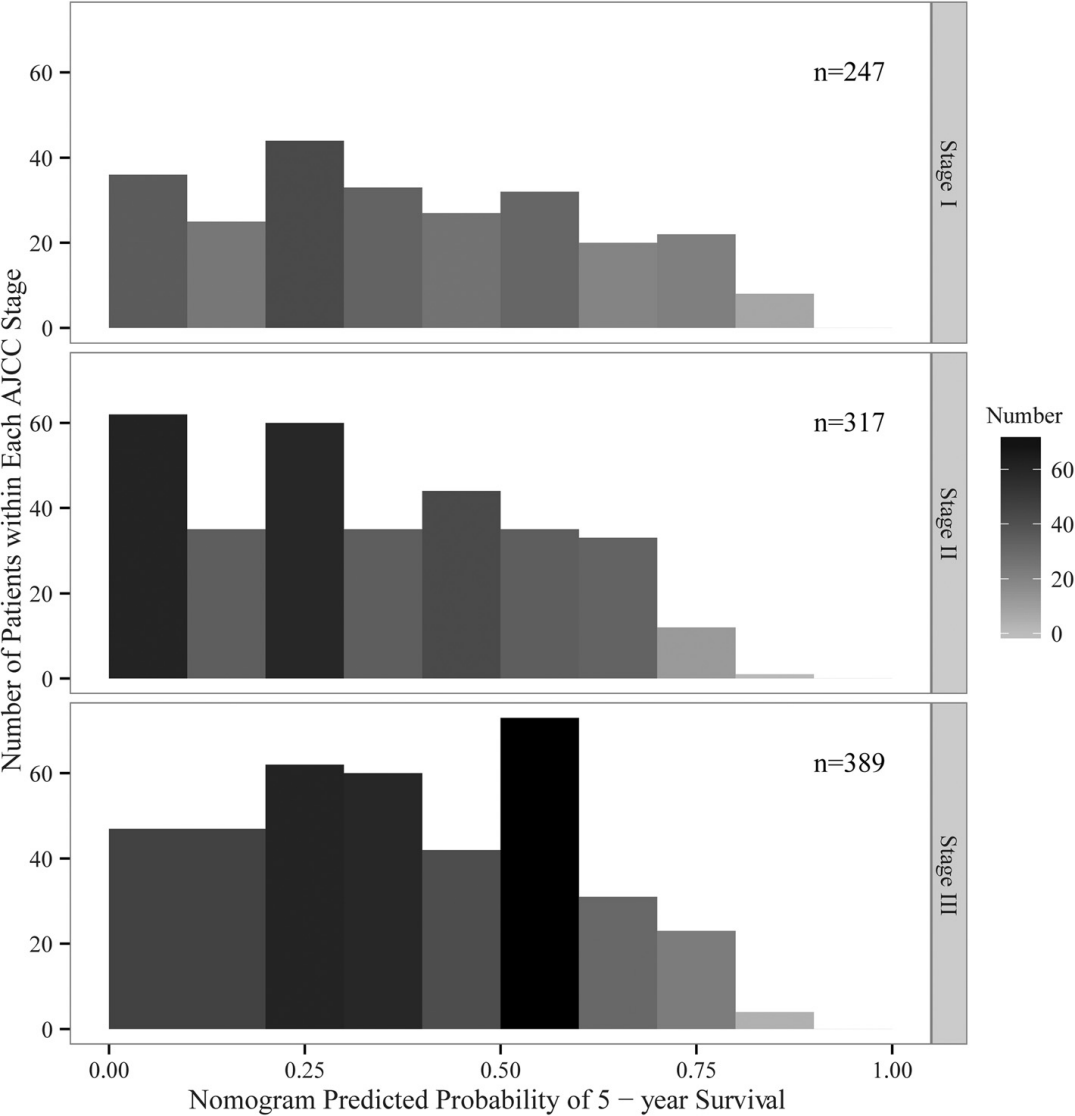
Predicted 5-year overall survival probability by AJCC TNM stage. A wide range of predicted survival could be identified in each TNM stage

Figure 6 provides the nomogram predicted 5-year overall survival probability sub-grouped by AJCC TNM AEG staging scheme. As a traditional staging system, the AJCC AEG stage was an important predictor of the overall survival probability. But other predictors used in our nomogram model added further information that was useful to make more accurate discrimination of the AEG patients’ prognosis. Overall, as mentioned above, the nomogram prediction model was also able to discriminate the high-risk, moderate-risk, and low-risk patients in a grouped survival analysis.

**Fig. 7:**
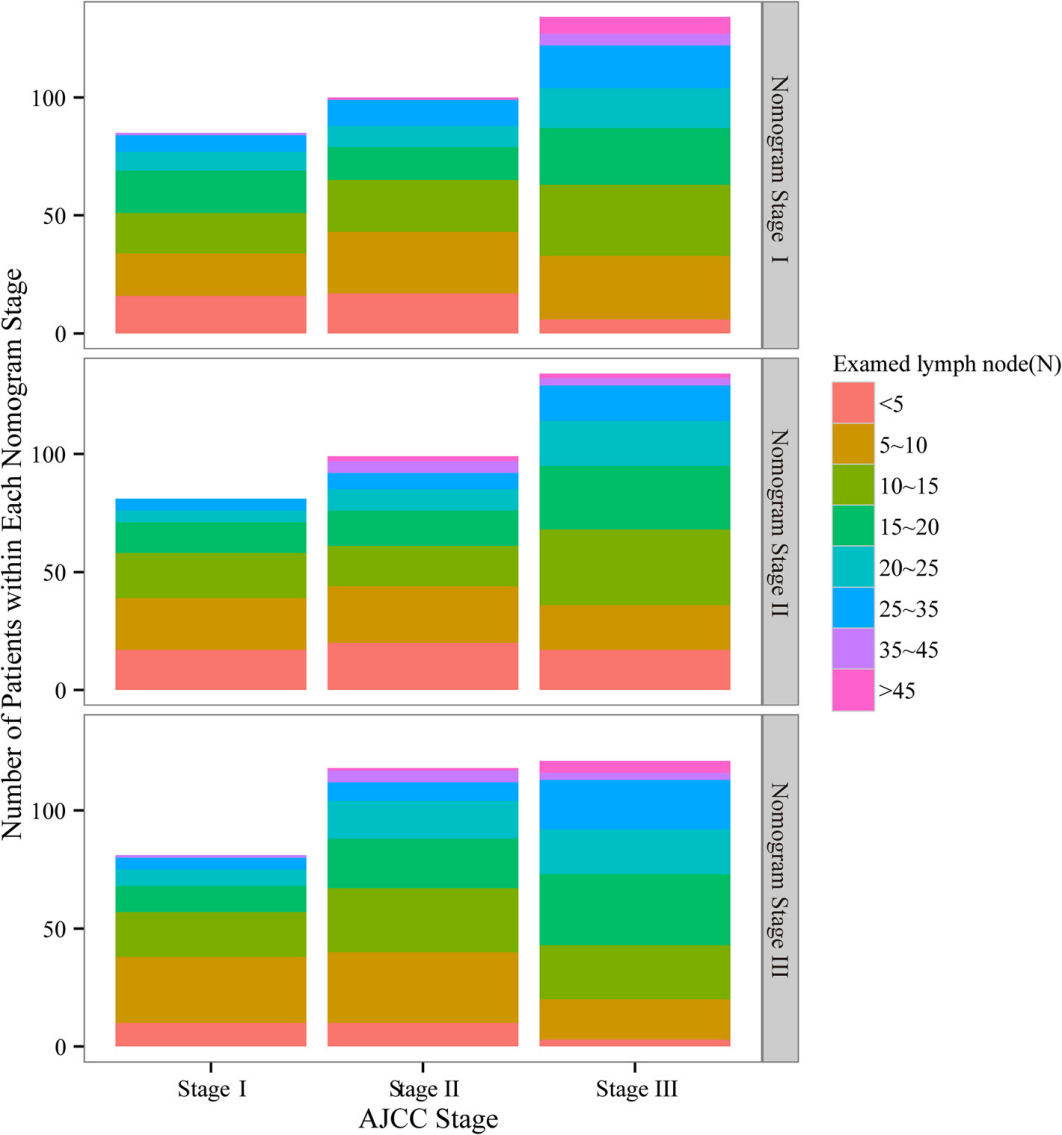
The distribution of the number of examined lymph nodes in different AJCC stages and nomogram stages

According to the multivariate analysis used in the nomogram, three of six prognostic factors were not used in the AJCC TNM AEG staging scheme. They were age, sex, and the number of examined lymph nodes during the lymphadenectomy and pathological examination. Among these three factors, the number of examined lymph nodes had the strongest prognostic weight. Figure 7 shows the distribution of the number of examined lymph nodes in different AJCC stages and nomogram stages.

## Discussion

As a junctional cancer, the adenocarcinoma of esophagogastric junction is always riding on the wave. Its classification, surgical approach, and the area of lymphadenectomy are still controversial. The 7th edition of AJCC TNM classification first defined the classification of AEG. If a tumor has an epicenter within 5 cm of the EGJ and extension into the esophagus, it would be classified and staged according to the esophageal scheme [5]. In previous studies, the Siewert type I (adenocarcinoma of the distal esophagus with the epicenter located within 1 and 5 cm above the anatomic EGJ) AEG was staged according to the esophageal scheme, while Siewert type II (adenocarcinoma of the cardia with the tumor epicenter within 1 cm above and 2 cm below the EGJ) and type III (adenocarcinoma with the tumor epicenter between 2 and 5 cm below the EGJ) were staged in terms of gastric scheme. AEG might have different biological properties compared with genuine gastric cancer and genuine esophageal cancer [15]. Therefore, further studies were required in order to ascertain if the latest scheme is suitable or not. In our nomogram system, the three prognostic factors (depth of invasion, metastasized lymph nodes, and pathological grade) which were also used in the AJCC staging system were assigned scores of different scales instead of permutations and combinations staging method used in AJCC staging system. Also, we classified the metastasized lymph nodes into five subgroups by considering both the gastric and esophageal cancer schemes. Furthermore, other latent prognostic variants, especially the number of examined lymph nodes, were taken into account in order to assess the overall survival.

The number of total examined lymph nodes from lymphadenectomy and the metastatic lymph node ratio (the proportion of metastasized lymph nodes of total examined lymph nodes) were identified as significant factors that affect the overall survival of patients with gastrointestinal cancer [16–19]. Resection of more lymph nodes could lead to a better overall survival and a more accurate survival estimation [20]. For AEGs, the different surgical procedures might result in different lymphadenectomy. Previous studies showed that transhiatal approach was chosen more frequently for AEGs of early AJCC stage, while the transthoracic approach for AEGs of advanced AJCC stage. Meanwhile, meta-analysis indicated that more lymph nodes were retrieved from AEGs via the transthoracic approach [21]. However, several recent studies did not achieve all the surgical quality criteria, especially the yields of lymphadenectomy. This might be due to particular anatomical position of AEG. Also, it was hard to conclude which procedure of lymphadenectomy was favorable. Nevertheless, we found a close correlation between the number of resected lymph nodes and overall survival. It was our opinion that the extent of lymphadenectomy is closely related with the overall survival of patients with AEG. Thus, we should estimate the personal survival of AEG patients in consideration of the total examined lymph nodes. This not only requires our surgeons to perform an adequate lymphadenectomy but also demands the pathologists to do their best in searching for lymph nodes.

After the combination of two more demographic characteristics (age and sex) with the four clinic factors (invasion depth, examined lymph nodes, metastatic lymph nodes and histological grade), the six factors calculated in nomogram had made a more accurate personal overall survival estimation of AEG patients. The prognostic nomogram directly quantified patient risk based on the variant prognostic factors without forming risk groups which was more favorable than the AJCC TNM classification estimated by C-index [22]. As we could see from Fig. 6, three AJCC TNM groups produced almost the same range of nomogram predicted survival. This indicated that the traditional AJCC classification might cause some bias when we predicted the personal overall survival. One study from Memorial Sloan Kettering Cancer Center (MSKCC) developed a postoperative nomogram for disease specific survival after curative surgery. But they mixed the AEG data with the gastric adenocarcinomas [23]. This would result in some inaccurate estimation of AEG. Meanwhile, another study from Netherlands calculated the nomogram score of patients with Siewert type I and type II [24]. Also, these two well-constructed nomogram scoring system were all from Western countries. It was uncertain whether they could be used to predict patients’ outcome of the East Asian countries. In our study, we used the data from SEER database which was constituted of patients most from Western countries to establish the training dataset and we used the patients from China to validate the nomogram scores generated from the training set. The result manifested that the two datasets displayed equal effectiveness in predicting personal overall survival.

## Conclusions

We developed and externally validated a nomogram predicting overall survival of AEG patients based on a Western and an Eastern database. The nomogram provided significantly better discrimination than the traditional AJCC TNM classification of AEGs and also provided an individualized prediction of the survival.

## Additional files

Additional file 1: Figure S1. Kaplan-Meier survival curves of AJCC TNM stages of non-metastasis patients with AEG in the validation set. Additional file 2: Figure S2. Kaplan-Meier survival curves of nomogram trisection stages of non-metastasis patients with AEG in the validation set.

## Abbreviations

AEG: adenocarcinomas of the esophagogastric junction
AJCC: American Joint Committee on Cancer
TNM: tumor-node-metastasis
EGJ: esophagogastric junction
MSKCC: Memorial Sloan Kettering Cancer Center
SEER: Surveillance, Epidemiology, and End Results Program
UICC: Union for International Cancer Control
