# Supplementary figures and images for: Nomogram predicted survival of patients with adenocarcinoma of esophagogastric junction
Source: World J Surg Oncol. 2015 Jun 10;13:197. doi: 10.1186/s12957-015-0613-7 (PMC4465317; doi:10.1186/s12957-015-0613-7)

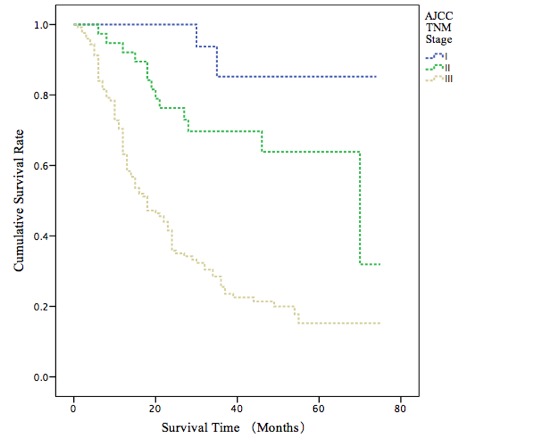

Supplement: Additional file 1: Figure S1. — Kaplan-Meier survival curves of AJCC TNM stages of non-metastasis patients with AEG in the validation set. [file 12957_2015_613_MOESM1_ESM.jpg]

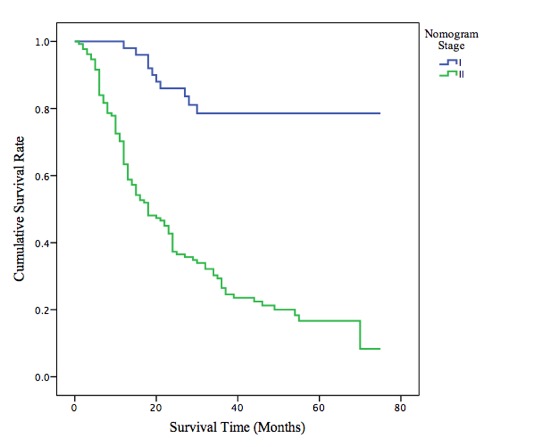

Supplement: Additional file 2: Figure S2. — Kaplan-Meier survival curves of nomogram trisection stages of non-metastasis patients with AEG in the validation set. [file 12957_2015_613_MOESM2_ESM.jpg]
